# Molecular Mechanisms of Proteinuria in Focal Segmental Glomerulosclerosis

**DOI:** 10.3389/fmed.2018.00098

**Published:** 2018-04-16

**Authors:** Yumeng Wen, Sapna Shah, Kirk N. Campbell

**Affiliations:** Division of Nephrology, Department of Medicine, Icahn School of Medicine at Mount Sinai, New York, NY, United States

**Keywords:** podocyte, focal segmental glomerulosclerosis, HIVAN, soluble urokinase-type plasminogen activator receptor, podocin

## Abstract

Focal segmental glomerulosclerosis (FSGS) is the most common primary glomerular disease resulting in end-stage renal disease in the USA and is increasing in prevalence worldwide. It is a diverse clinical entity with idiopathic, genetic, metabolic, infectious, and other causes that culminate in a characteristic histologic pattern of injury. Proteinuria is a hallmark of FSGS as well as other primary and secondary glomerular disorders. The magnitude of proteinuria at disease onset and during treatment has prognostic implications for renal survival as well as associated cardiovascular morbidity and mortality. Significant advances over the last two decades have shed light on the molecular architecture of the glomerular filtration barrier. The podocyte is the target cell for injury in FSGS. A growing list of disease-causing gene mutations encoding proteins that regulate podocyte survival and homeostasis has been identified in FSGS patients. Several pathogenic and regulatory pathways have been uncovered that result in proteinuria in rodent models and human FSGS. The recurrence of proteinuria and FSGS after kidney transplantation is supporting evidence for the role of a circulating permeability factor in disease pathogenesis. These advances reviewed herein have significant implications for disease classification and therapeutic drug development for FSGS.

## Introduction

The glomerular filtration barrier is composed of fenestrated endothelial cells, the glomerular basement membrane (GBM), and podocytes, terminally differentiated epithelial cells connected by interposed slit diaphragms between actin-rich foot processes (1). Seminal studies by Farquhar and Palade highlight the role of the GBM in excluding molecules of the size and negative charge of albumin from the urine under normal conditions (2). Though proteinuria can result from injury to any of these components, experimental nephrosis was initially described as mainly affecting the visceral epithelium with loss of podocyte foot processes, reduction and modification of the “urinary slits” (interposed slit diaphragm), and intracellular accumulation of vacuoles and protein absorption droplets (3, 4). Positional cloning of the NPHS1 gene encoding nephrin, a vital component of the slit diaphragm, was a landmark event that initiated the era of molecular discovery of the complex molecular architecture of the podocyte (5). Wiggins et al. performed elegant studies showing that in a diphtheria toxin rat model, podocyte loss of more than 20% results in a low-level-sustained proteinuria. Loss greater than 40% directly causes a high-level proteinuria, a decreased renal function, and lesions of focal segmental glomerulosclerosis (FSGS) (6). Podocyte loss triggers a cascade involving tuft adhesions of bare areas of GBM to Bowman’s capsule, a nidus for sclerosis development that can progress to segmental sclerosis (7). Rather than a disease, FSGS should be considered a histologic pattern of glomerular injury resulting from podocyte loss. It describes not only primary podocyte injury but also a lesion that occurs as a secondary process such as hypertensive and diabetic nephropathy (DN). Clinically and genetically heterogeneous, FSGS is characterized by segmental sclerosis of the glomerular capillary tuft, with or without deposition of IgM and complement C3 (8). The pathogenesis of FSGS is multifactorial, thereby complicating classification efforts and therapeutic approaches. The contribution of circulating permeability factors has been suggested for four decades with the recurrence of FSGS after kidney transplantation potentially occurring within hours (9, 10). Separately, many genes identified in patients with familial and sporadic FSGS encode essential podocyte proteins (11). Viral nephritides, HIV in particular, has been associated with collapsing FSGS (12).

This review discusses the knowledge to date of the underlying pathogenesis of podocyte injury leading to proteinuria and FSGS. We focus on the contributions of putative circulating factors, gene mutations, and viral infections.

## Circulating Factors

The high recurrence rate of FSGS post transplant (estimated at 30–40%) suggests the presence of a circulating factor in recipients. A pivotal study by Savin et al. showed that plasma from patients with recurrent FSGS could increase the glomerular permeability to albumin in an *in vitro* assay. Plasmapheresis was associated with a reduced proteinuria as well as a reduced glomerular permeability (13). Gallon presented an interesting case of a kidney-transplant patient with recurrent FSGS (14). The allograft kidney regained function with a reduction in proteinuria after the graft was removed and re-transplanted to another patient whose ESRD was due to diabetes mellitus. In addition, an infant born to a mother with primary FSGS developed transient proteinuria, suggesting a circulating factor crossing the placenta leading to proteinuria (15). These studies well illustrate the existence of a circulating factor, but the nature and source of such a factor remained unclear. Several putative factors have been proposed including soluble urokinase-type plasminogen activator receptor (suPAR), CLC-1, Apolipoprotein A1, active protease, and anti-CD40.

### Soluble Urokinase-Type Plasminogen Activator Receptor

Of the putative factors, the role of urokinase-type plasminogen activator receptor (uPAR) and its soluble form (suPAR) have been the most studied (16, 17). uPAR is a glycosylphosphatidylinositol (GPI)-anchored three-domain (DI, DII, and DIII) protein. Apart from being a receptor for urokinase, uPAR also forms signaling complexes with various transmembrane proteins including integrin and is involved in non-proteolytic pathways. The soluble form suPAR can be released by the cleavage of GPI anchor. In Wei’s study, uPAR was upregulated in the glomeruli from both DN and FSGS. Using uPAR knockout (Plaur^−/−^) mice model, they showed that lipopolysaccharide (LPS)-induced proteinuria was uPAR-mediated, and uPAR upregulation following LPS stimulation led to αvβ3 integrin activation, with subsequent podocyte foot process effacement, increased podocyte motility, and proteinuria (16). The same group subsequently revealed the role of suPAR as a potential pathogenic factor involved in podocyte injury (17). Recombinant suPAR and serum from patients with FSGS recurrence induced αvβ3 integrin activation both *in vitro* and *in vivo*. In uPAR-knockout (Plaur^−/−^) mice, the supraphysiologic level of endogenous suPAR and exogenous suPAR injection induced αvβ3 integrin activation, resulting in podocyte injury and proteinuria. A recent study (18) has shed light on the source of suPAR production. Myeloid progenitor Gr-1^lo^ cell expansion along with suPAR upregulation and proteinuria was observed in different rodent models for proteinuric kidney disease including DN and nephrotoxic serum nephritis. Incubating Gr-1^lo^ − Sca-1 + myeloid progenitor cells with LPS led to uPAR expression and suPAR secretion, and injecting these myeloid progenitor cells could induce proteinuria in NSG mice (deficient in mature lymphocytes and natural killer cells). To translate their findings to human disease, the investigators introduced whole peripheral blood mononuclear cells (human hematopoietic cells), drawn from individuals with recurrent FSGS and injected into NSG mice. This approach led to Gr-1^lo^ cell expansion, suPAR upregulation, and proteinuria. However, CD34+-depleted PBMC from these patients failed to induce any proteinuria. This process was distinct from GVHD pathophysiology since T cell-depleted PBMC yielded similar results. Though human homologs of Gr-1^lo^ − Sca-1 + myeloid progenitor cells have not been identified given the lack of Gr-1 or Sca-1 antigen, these findings implicate a common upstream pathway relevant to general CKD. The same investigators recently linked APOL1-risk alleles to the suPAR–β3 integrin activation pathway (19). In this study, G1/G2-risk alleles were demonstrated to activate β3 integrin synergistically with suPAR, inducing autophagosome formation in podocytes and leading to foot process effacement, podocyte detachment, and proteinuria.

These findings have not been without controversy. Conflicting studies have shown that suPAR does not distinguish FSGS from other causes of nephrotic syndrome and that suPAR expression may be nonspecifically increased in the setting of a low eGFR (20–22). These findings have raised questions about the reliability of current ELISA methodology as a diagnostic biomarker for FSGS (23). It is likely that suPAR’s utility is confined to patients with a preserved eGFR. This is supported by the fact that elevated suPAR levels predicted future CKD development in the 3683 subject Emory Cardiovascular Biobank (24). The fact that suPAR was associated with both proteinuric and non-proteinuric CKD in this cohort also highlights the need for additional mechanistic studies on suPAR targets, production, and mechanism of action.

#### Other Proposed Circulating Permeability Factors

##### Cardiotrophin-Like Cytokine Factor 1 (CLCF1)

Proteomic analysis by liquid chromatography tandem mass spectrometry of recurrent FSGS plasma fractions that induce proteinuria in rats and enhance glomerular permeability to albumin led to the identification of CLCF1 (25–27). CLCF1 is a member of the IL-6 cytokine family and is secreted into the circulation as a heterodimer with either cytokine receptor-like factor 1 (CRLF1) or soluble ciliary neurotrophic factor receptor alpha. This heterodimeric coexpression is essential for efficient CLF1 secretion (28, 29). It has been demonstrated that CLCF1 increased glomerular permeability to albumin in a specific manner with anti-CLCF1 monoclonal antibody, blocking this effect and attenuating the effect of FSGS serum (30). CLCF1 appeared to be JAK/STAT dependent with both JAK2 and STAT3 inhibition blocking the ability of CLCF1 to increase glomerular permeability to albumin. Interestingly, heterodimeric CLCF1–CRLF1 also had an inhibitory effect on CLCF1 and FSGS-induced albumin permeability (30). This would suggest that while monomeric CLCF1 is pathogenic, heterodimeric CLCF1 is protective. The study also highlights the need for further exploration of the JAK–STAT pathway in FSGS pathogenesis.

##### Anti-CD40 Antibody

Recently, Delville et al. (31) screened 9,000 antigens in pretransplant sera from 64 patients with or without recurrent FSGS, compared to 34 non-FSGS CKD patients. A panel of antibodies (CD40, PTPRO, CGB5, FAS, P2RY11, SNRPB2, and APOL2) could predict posttransplant FSGS recurrence with 92% accuracy. Pretransplant elevation anti-CD40 antibody alone had the best accuracy of 78% in predicting FSGS recurrence. Anti-CD40 antibody caused podocyte injury both in cultured human podocytes and in wild-type mice, and the formation of this antibody may be associated with altered immunogenicity of the CD40 protein in serum among patients with recurrent FSGS. Proteinuria was further enhanced by co-injecting full-length suPAR into wild mice, suggesting that uPAR–αvβ3 signaling pathway may also be involved in anti-CD40 antibody-induced podocyte injury (31). This study was limited by a small sample size with validation required in other recurrent FSGS cohorts.

##### Apolipoprotein A-I

Proteomic analysis of plasma and urine samples performed on patients with recurrent FSGS post transplant identified a high-molecular-weight form of Apolipoprotein A-I, named as ApoA-Ib in 93% of recurrent FSGS urines compared with <5% of those without recurrence, patients with non- FSGS proteinuric diseases, or patients transplanted for familial FSGS. Urinary ApoA-Ib had a sensitivity of 92.8% and a specificity of 98.1% for identifying FSGS relapse (32). Urinary ApoA-Ib warrants additional investigation as a biomarker of FSGS recurrence post transplant. It remains unclear whether it is a cause or a consequence of FSGS recurrence. A potential pathogenic role in causing podocyte injury has not been established.

##### Active Proteases

There is evidence that plasma proteases may have a pathogenic role in recurrent FSGS. Vasodilator-stimulated phosphoprotein (VASP), a molecule involved in actin cytoskeleton organization is phosphorylated in response to exchange plasma from 10 patients with recurrent FSGS (33). VASP phosphorylation was associated with pathogenic-enhanced podocyte motility. Protease inhibitory drug cocktails and silencing of the protease-activated receptor-1 led to the loss of VASP phosphorylation. The source of increased proteases or whether they are enhanced by protease inhibitor loss remains unclear.

## Genetic Etiologies of FSGS

Beginning with nephrin, the positional cloning of patients and relatives with familial FSGS has enabled the identification of numerous disease-causing podocyte genes (Table 1). They encode podocyte proteins localized to the cell membrane (TRPC6), nucleus (WT1), mitochondria (COQ2 and COQ6), lysosomes (LIMP2), and cytosol (PLCE1) (34–43). The most common mutations encode actin cytoskeletal (INF2, ACTN4, MYO1E) and slit diaphragm (NPHS1, NPHS2, CD2AP) (5, 44–49) proteins, thereby highlighting the essential role of these structures in the maintenance of the glomerular filtration barrier. Mutations in the ACTN4 gene are all in the actin-binding domain of the encoded protein and have a much higher affinity for actin filaments than of the wild-type protein (50). This gain of function produces a rigid cytoskeleton more susceptible to stress and actin network breaking. INF2, a member of the formin family, also regulates the actin cytoskeleton but in a different way. INF2 variants occur in the diaphanous inhibitory domain that is essential in the inhibition of Rho activation (51). Unchecked Rho signaling has therefore been suggested as a potential etiology for podocyte injury in INF2 mutation (51). Indeed, the activation of RhoA in podocytes has been shown to lead to albuminuria, podocyte foot process effacement, and histologic lesions of FSGS (52).

**Table 1:** Gene mutations linked to focal segmental glomerulosclerosis (FSGS).

Gene	Protein	Gene locus	Mode of inheritance	Phenotype
**Slit diaphragm**				
NPHS1	Nephrin	19q13.1	AR	Congenital nephrotic syndrome Finnish type, sporadic FSGS, nephrotic syndrome
NPHS2	Podocin	1q25.31	AR	Minimal change nephropathy, FSGS
CD2AP	CD2-associated protein	6p12	AD/AR	AD or AR sporadic adult-onset FSGS
TRPC6	TRPC6	11q22.1	AD	Adult-onset FSGS
**Actin cytoskeleton and cytosol**				
ACTN4	Alpha-actinin 4	19q13.1	AD	Adult-onset FSGS
INF2	Inverted formin 2	14q32.33	AD	Adult-onset FSGS, Charcot–Marie tooth disease
MYO1E	Myosin 1E	15q22.2	AR	Early-onset autosomal-recessive FSGS
ARHGAP24	Arhgap24 (RhoGAP)	4q22.1	AD	Adolescent-onset FSGS
ARHGDIA	Arhgdia	17q25.3	AR	Early-onset nephrotic syndrome or FSGS
PLCE1	Phospholipase C epsilon 1	10q23.33	AR	Early-onset diffuse mesangial sclerosis and FSGS
PTPRO	Receptor-type tyrosine-protein phosphatase-0	12p12.3 by	AR	Childhood FSGS
**Syndromic conditions**				
WT1	Wilms’ tumor 1	11p13	AD	Diffuse mesangial sclerosis and FSGS, Frasier or Denys–Drash syndrome; GU abnormalities
LXMB1	Lim homeobox transcription factor 1B	9q31.1	AD	FSGS, dystrophic nails, absent or malformed patella
tRNA^LEU^		Mitochondrial	Maternal	FSGS, tubulointerstitial nephritis
COQ2	Coenzyme Q2 homolog, prenyltransferase	4q21.22	AR	FSGS, neurologic, and muscle abnormalities
COQ6	Ubiquinone biosynthesis monooxygenase COQ6	14q24.3	AR	FSGS, deafness
ITGB4	Integrin B4	17q25.1	AR	FSGS, epidermolysis bullosa
PDSS2	Decaprenyl diphosphate synthase subunit 2	6q21		FSGS or collapsing FSGS
**Glomerular basement membrane**				
CD151	CD151 Antigen	11p15.5		Early FSGS, deafness, β-thalassemia
CUBN	Cubilin	10p13	AR	Chronic glomerulosclerosis, FSGS, or HIV-associated nephropathy
LAMB2	Laminin beta 2	3p21	AR	Isolated nephrotic syndrome as part of Pierson syndrome
**Function unknown**				
APOL1	Apolipoprotein L1	22q12.3		FSGS, hypertensive-associated kidney disease and HIV nephropathy

The Rho family small GTPases (RhoA, Rac1, and Cdc42) are essential in actin cytoskeletal dynamics, cell morphology, motility, and adhesion. While overactive RhoA can be deleterious, there is an emerging consensus that a relative predominance of RhoA activity relative to Rac1/Cdc42 favors a more stationary podocyte with intact foot processes (53, 54). Conversely, Cdc42/Rac1 activation is associated with a more motile podocyte phenotype and foot process retraction. Loss of function mutations in the Arhgap24 gene associated with familial FSGS cause increased levels of active Rac1 and Cdc42 and increased podocyte motility (55). Likewise, ARHGDIA mutations in patients with steroid-resistant nephrotic syndrome increase active GTP-bound Rac1 and Cdc42 again resulting in increased podocyte motility reversed with Rac1 inhibitors (56). The slit diaphragm is also intricately connected to podocyte actin cytoskeletal dynamics. CD2AP and nephrin are direct binding partners with CD2AP interacting with actin, cortactin, and the alpha-actinin modulating protein synaptopodin (57, 58). Nck adaptor proteins link nephrin to the actin cytoskeleton (59). The cytoplasmic domain of nephrin contains six conserved tyrosine residues that when phosphorylated facilitates binding to the SH2 domains of Nck, leading to actin polymerization (60). Upregulated nephrin tyrosine phosphorylation has been described in glomerular injury (61), but the involved tyrosine kinase remains undefined.

The onset of proteinuria is variable in genetic disease with autosomal-recessive etiologies generally manifesting clinically in childhood and autosomal-dominant traits in adulthood. Commercially available genetic testing is most likely to identify known disease-causing variants in infants and patients with familial and syndromic disease (62). Exomic and genomic sequencing have the potential to uncover rare novel gene variants even in sporadic disease, but confirming causation typically requires experimental modeling with cell- and animal-based models. Identification of disease-causing gene mutations in FSGS has prognostic consequences since these patients are less likely to respond to steroid and calcineurin inhibitor therapy (63). They should perhaps be prioritized for kidney transplantation since disease recurrence is lower (64). Caution is necessary in the clinical application of genetic data, however. It is estimated that only 2.9% of US patients with nephrotic syndrome have a monogenic form of the disease and there is a risk in attributing a pathogenic role to identified variants that could be noncausal (65, 66).

### Apolipoprotein L1 (APOL1)

Focal segmental glomerulosclerosis occurs with a higher frequency in African Americans largely due to variations in the Apol1 gene encoding APOL1 (67). APOL1 is a plasma factor that lyses the parasite *Trypanosoma b. brucei*. Over time, the parasite evolved into a serum resistance-associated protein (SRA) containing *T. brucei* rhodesiense that cause African sleeping sickness and capable of inactivating and evading Apol1. G1 and G2 Apol1 gene variants evade the SRA and are active against *T. brucei* rhodesiense. In this sense, this is analogous to malaria where the sickle trait is protective against parasitic infection but causes a hemoglobinopathy. Located on chromosome 22, the G1 and G2 alleles confer an increased risk for developing FSGS, hypertensive-associated kidney disease, and HIV nephropathy (68). In some population-based studies, it has been shown that APOLI1 variants conferred a 17-fold risk of FSGS and a 29-fold risk of HIVAN (69). Interestingly, 9 of 10 (90%) African American patients with collapsing FSGS in one cohort were noted to have at least one APOL1-risk allele (70). Several recently published studies have increased the understanding of how APOL1 G1 and G2 variants induce podocyte injury. Mice with podocyte-specific expression of either allele develop foot process effacement, proteinuria, and azotemia (71). These variants were found to interfere with endosomal trafficking and autophagic flux within podocytes leading to inflammatory-mediated cell death and glomerulosclerosis. Defects in autophagy have been previously shown to induce podocyte injury, proteinuria, and FSGS (72, 73), and manipulation of autophagic pathways could form the basis for therapeutic targeting of Apol1. These findings were distinct from those published by another group in which podocyte-specific APOL1-G2 transgenic mice developed preeclampsia but not kidney disease (74). The difference could be explained by the level-lower APOL1 levels obtained in the latter model. Other potential mechanisms have been implicated in APOL1-induced podocyte injury based on *in vitro* studies. In a human embryonic kidney cell, Tet-on system expression of G1 and G2 variants enhanced efflux of extracellular potassium and subsequent activation of stress-activated protein kinases (SAPK), p38 MAPK, and JNK. Interestingly, cytotoxicity was abrogated by SAPK inhibition and inhibition of K+ efflux (75). Apol1 has also been postulated to be involved in apoptosis, mitochondrial dysfunction, and energy depletion in other cell-based studies (76–78). Merely carrying high-risk APOL1 variants is not sufficient to cause kidney disease, and significant attention is currently being directed toward identifying second hits in the form of environmental and infectious triggers that promote disease development. The identification of patients lacking APOL1 with a normal phenotype increases the prospect that it can be therapeutically targeted.

## Viral-Associated Disease

Podocytes can be injured by viruses either directly or by inflammatory cytokine-mediated targeting of podocyte receptors. In this context, HIV has been best studied. The renal expression of HIV genes has long been recognized as a central role in promoting HIVAN pathogenesis (79). HIV infects podocytes, tubular epithelial cells, infiltrating lymphocytes, as well as macrophages. Early studies of animal models, including the classic transgenic Tg26 mouse lines, studies on reciprocal transplantation between Tg26 and wild-type mice as well as podocyte-specific expression of HIV genes have confirmed the pathogenic role for local HIV gene expression in the kidney (80). Podocyte-predominant infection has been associated with podocyte injury, dedifferentiation, and more rapid loss of kidney function, suggestive of an HIV-driven podocyte injury as a primary pathogenic pathway in HIVAN (81). Moreover, the kidney has been shown to be one of the reservoirs for HIV, allowing active viral replication in this compartment apart from blood (82, 83). In HIVAN and collapsing forms of primary FSGS, injured podocytes were found to revert to a developmental program that includes downregulation of cyclin kinase inhibitors, entry into the cell cycle, upregulation of proliferation maker, and loss of mature phenotypic makers, including CD10/CALLA, C3b receptor, GLEPP-1, podocalyxin, synaptopodin, and importantly, WT1 (84–88). This process, termed podocyte dysregulation or podocyte dedifferentiation, is not observed in other proteinuric kidney disease marked by podocyte injury along with foot process effacement, such as minimal change disease and membranous nephropathy.

Cip/Kip family of cyclin kinase inhibitors acts in both G1 and S phases, while p21 also inhibits G2/M phase complexes. In healthy podocytes, p27 and p57 are expressed but p21 is suppressed (85). However, in patients with HIVAN or idiopathic collapsing FSGS, the expression of p27 and p57 is significantly suppressed, along with an increase in pro-mitotic cyclins (cyclin A), an increase in proliferation marker ki-67, and an expression of p21 (84, 85). Interestingly, the decrease in p27 and p57 was also found in histologically normal glomeruli, suggestive of a decrease of anti-mitotic signals preceding morphological change. Moreover, administering cyclin kinase inhibitors is associated with a restoration of normal podocyte phenotype and attenuated HIVAN presentation without any suppression of HIV gene expression (89, 90). Podocyte dedifferentiation is accompanied by the proliferation of parietal epithelial cells (PECs), which express parietal cell marker such as CK8 and PAX2 and lack podocyte markers. Cell bridges to the CK8-positive parietal lining could be observed without any evidence of coexpression of podocyte markers. These findings suggest that glomerular PECs provide a niche for podocyte progenitor cells (12, 91, 92).

Novel mechanisms of HIV-mediated kidney injury have been recently uncovered. Mammalian target of rapamycin (mTOR) has been found to be critical for p53-induced oxidative cell injury with mTOR inhibition protecting against HIV-induced podocyte apoptosis (93). The protective function of mTOR inhibition in HIVAN could be due to the regulation of microRNAs since rapamycin treatment reverses the downregulation of miR99a, miR-100a, miR-199a, miR-200a, miR-200b, miR-200c, miR-429, and miR-141 caused by HIV infection of human podocytes in culture (94). HIV also compromises the podocyte actin cytoskeleton through downregulation of the vitamin D receptor with associated enhanced deleterious intracellular angiotensin II and cathepsin L expression (95).

### Other Viral-Associated FSGS

Collapsing FSGS has long been associated with parvovirus B19 infection (96, 97). The prevalence of parvovirus DNA infection within renal tissue is associated with idiopathic FSGS and collapsing FSGS compared with other diseases (98). Using *in situ* hybridization techniques, parvovirus B19 DNA was localized at endothelial cells and both visceral and PECs. However, whether this viral infection results in podocyte dysfunction is still a matter of debate (97). CMV has been associated with FSGS (99), but it remains unclear whether this virus directly infects podocytes. The detection of tubuloreticular inclusions in glomerular endothelial cells in CMV infection suggests a pathogenic role for interferon stimulation (100). Susceptible patients may have identifiable genetic risk factors. Indeed, a cohort of patients receiving interferon treatment who developed collapsing FSGS was reported to carry high-risk ApoL1 genotypes. Interferon α, β, and γ treatment of cultured podocytes increased APOL1 expression (101). Upstream of interferons, Toll-like receptor 3 (TLR3) was also shown here to signal through TBK1, NFkB, and JAK kinases in an interferon-independent manner to also increase APOL1 expression where the TLR3 agonist polyl:C promotes the binding of transcription factor IRF1/2 and STAT2 at the APOL1 transcription start site (101). These findings highlight the complex interplay between viruses, antiviral defenses, and the APOL1 genotype in the development of HIV and non-HIV-mediated viral FSGS.

## Decreased Nephron Mass

Low birth weight individuals have long been known to be susceptible to a reduced nephron number and resulting abnormal hemodynamic stress on remaining nephrons (102, 103). Low birth weight is more commonly seen in FSGS patients than in the general population (104). Proteinuria has also been recognized in patients with unilateral renal agenesis where the remaining kidney has a reduced nephron number (105). A decreased nephron mass results in glomerular hypertension and hyperfiltration that increase mechanical stretch and injury to podocytes (106, 107). Glomerular hyperfiltration has also been associated with the pathogenesis of obesity-related FSGS (108). Here as with a decreased nephron mass, the activation of the renin–angiotensin–aldosterone system, the upregulation of TGFβ, and glomerulomegaly contribute to the vicious cycle that leads to proteinuria and FSGS (109, 110). Angiotensin blockers are the mainstay of treatment for patients with proteinuria due to hyperfiltration. There is some evidence that bariatric surgery can normalize proteinuria in patients with obesity-related glomerular disease (111).

## Targeted Therapy

**Figure 1:**
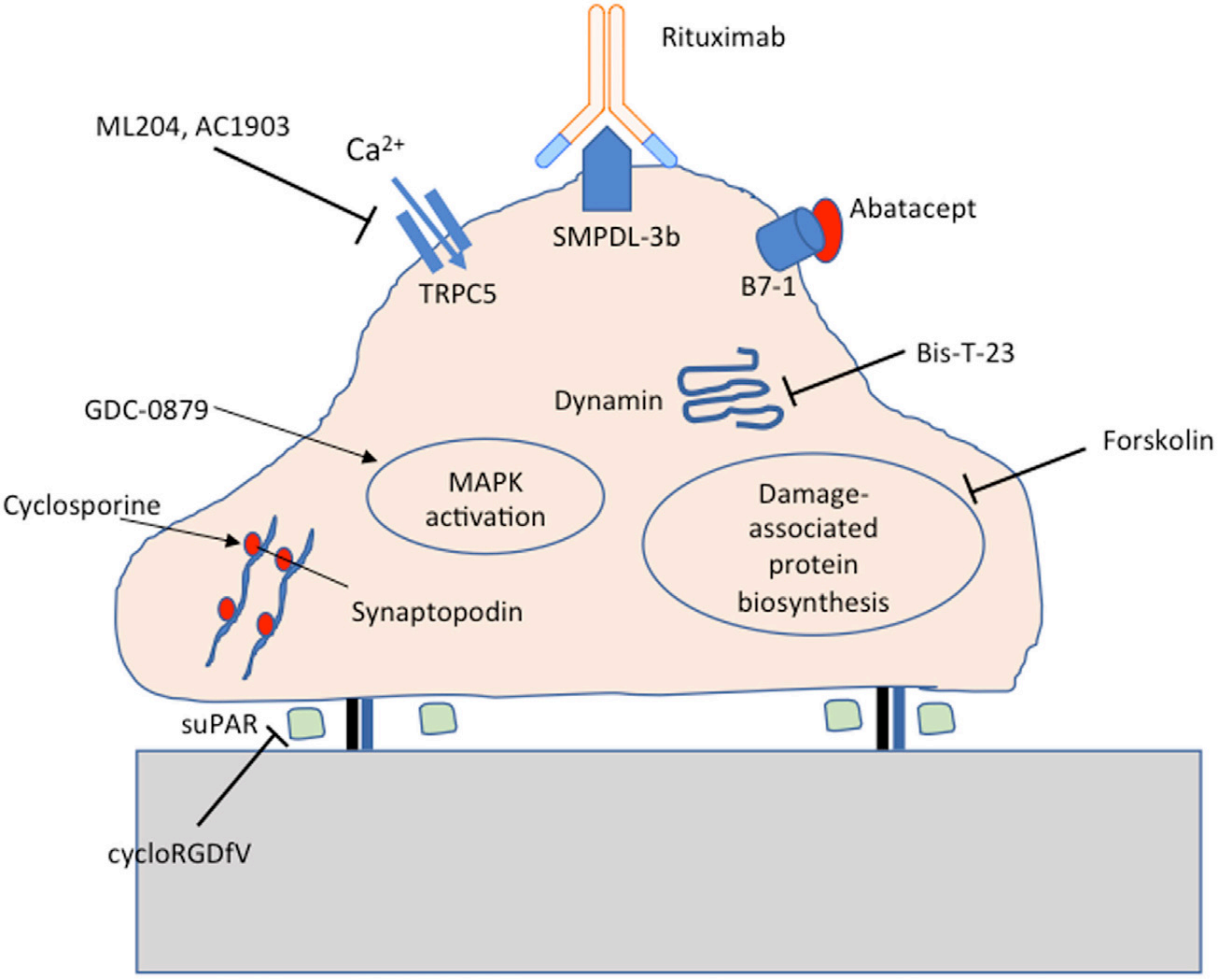
Targeted therapeutics for podocyte protection. Cyclosporine protects synaptopodin from cathepsin-mediated cleavage; cycloRGDfV inhibits soluble urokinase-type plasminogen activator receptor (suPAR) mediated β3 integrin activation; GDC-0879 promotes protective MAPK activation; ML204 and AC1903 inhibit TRPC5 ion channels; Rituximab preserves sphingomyelin phosphodiesterase acid-like 3b (SMPDL-3b) expression; Abatacept inhibits B7-1 mediated podocyte injury; Bis-T-23 promotes actin-dependent dynamin oligomerization; Forskolin inhibits damage-associated protein biosynthesis.

The identification of molecules essential for maintaining podocyte homeostasis has opened the door to targeted therapeutics for FSGS. Indeed, a number of repurposed drugs currently in clinical use have been shown to act directly on podocytes. Steroids protect podocytes from puromycin aminonucleoside-induced injury (112). Rituximab protects podocytes from injury by preserving sphingomyelin phosphodiesterase acid-like 3b (SMPDL-3b) expression (113). Cyclosporine stabilizes the actin-bundling protein synaptopodin, preventing cathepsin-mediated cleavage (114). Novel target-specific agents being tested at various stages of development include GDC-0879 (115), AC1903 (116), Abatacept (117), Bis-T-23 (118), and cycloRGDfV (17) as summarized in Figure 1.

## Conclusion

Podocyte injury due to circulating factors, gene mutations, infections, and many diverse etiologies culminate in the clinical manifestation of proteinuria and the histologic finding of FSGS. The evidence for a circulating permeability factor has been evolving in recent years, and various studies have suggested the pathogenic role of suPAR and other factors in the development of FSGS. The composition, origin, identity, and synergistic roles of circulating factor(s) remain unclear, representing an area of high unmet clinical need. Increasingly, podocyte-associated gene mutations are being identified in patients with FSGS. Expanded knowledge of the molecular architecture of the glomerular filtration barrier raises the prospect of a targeted therapeutic drug development.

HIV and other viral infections can induce podocyte injury and FSGS with an increasing interest in the enhanced susceptibility conferred by APOL1-risk alleles. The numerous divergent etiologies and pathogenic mechanisms with resulting variable response rates to therapy and recurrence post transplant speak to the need for a reclassification of the disease into more clinically relevant subtypes. Further exploration of the contributions of JAK/STAT, Rho-GTPase, and ApoL1 to podocyte injury and survival could enhance the quest for novel therapeutic agents.

## Author Contributions

YW, SS, and KC conceived the article contents, prepared the manuscript, and endorsed the final draft submitted.

## Conflict of Interest Statement

The authors declare that the research was conducted in the absence of any commercial or financial relationships that could be construed as a potential conflict of interest.
